# Rheology of Dispersions of High-Aspect-Ratio Nanofibers Assembled from Elastin-Like Double-Hydrophobic Polypeptides

**DOI:** 10.3390/ijms20246262

**Published:** 2019-12-12

**Authors:** Ayae Sugawara-Narutaki, Sawako Yasunaga, Yusuke Sugioka, Duc H. T. Le, Issei Kitamura, Jin Nakamura, Chikara Ohtsuki

**Affiliations:** 1 Department of Materials Chemistry, Graduate School of Engineering, Nagoya University, Furo-cho, Chikusa-ku, Nagoya 464-8603, Japan; sugioka.yusuke@h.mbox.nagoya-u.ac.jp (Y.S.); h.t.d.le@tue.nl (D.H.T.L.); nakamura@chembio.nagoya-u.ac.jp (J.N.); ohtsuki@chembio.nagoya-u.ac.jp (C.O.); 2 Department of Crystalline Materials Science, Graduate School of Engineering, Nagoya University, Furo-cho, Chikusa-ku, Nagoya 464-8603, Japan; sawako.yasunaga@gmail.com; 3 Department of Molecular and Macromolecular Chemistry, Graduate School of Engineering, Nagoya University, Furo-cho, Chikusa-ku, Nagoya 464-8603, Japan; kitamura.issei@c.mbox.nagoya-u.ac.jp

**Keywords:** elastin-like polypeptide, self-assembly, block copolymer, nanofiber, dispersion, rheology

## Abstract

Elastin-like polypeptides (ELPs) are promising candidates for fabricating tissue-engineering scaffolds that mimic the extracellular environment of elastic tissues. We have developed a “double-hydrophobic” block ELP, **GPG**, inspired by non-uniform distribution of two different hydrophobic domains in natural elastin. **GPG** has a block sequence of (VGGVG)_5_-(VPGXG)_25_-(VGGVG)_5_ that self-assembles to form nanofibers in water. Functional derivatives of **GPG** with appended amino acid motifs can also form nanofibers, a display of the block sequence’s robust self-assembling properties. However, how the block length affects fiber formation has never been clarified. This study focuses on the synthesis and characterization of a novel ELP, **GPPG**, in which the central sequence (VPGVG)_25_ is repeated twice by a short linker sequence. The self-assembly behavior and the resultant nanostructures of **GPG** and **GPPG** were when compared through circular dichroism spectroscopy, atomic force microscopy, and transmission electron microscopy. Dynamic rheology measurements revealed that the nanofiber dispersions of both **GPG** and **GPPG** at an extremely low concentration (0.034 wt%) exhibited solid-like behavior with storage modulus G′ > loss modulus G” over wide range of angular frequencies, which was most probably due to the high aspect ratio of the nanofibers that leads to the flocculation of nanofibers in the dispersion.

## 1. Introduction

Self-assembling peptides are promising candidates for the fabrication of tissue-engineering scaffolds, because their nanostructures resemble those of extracellular matrix (ECM) proteins, and their physicochemical and biological properties can be tailored by designing its amino acid sequences [1,2,3,4]. Elastin-like polypeptides (ELPs), which contain repetitive amino acid sequences that are found in elastin, an ECM protein abundant in elastic tissues, are among the self-assembling polypeptides that have been intensively explored for biomedical applications [5,6,7,8]. As ELPs are biocompatible and inherit their elastic recoil properties from natural elastin, they are considered to be rational materials for mimicking the extracellular environment of elastic tissues, such as lung, blood vessel, ligament, and skin [9,10,11,12]. Typical ELPs are comprised of (Val-Pro-Gly-Xaa-Gly)_n_ ((VPGXG)_n_), where Xaa is a guest amino acid residue with the exception of Pro [13]. They are soluble in cold water, but become insoluble and coacervate at above their transition temperature (*T*_t_), which can be precisely controlled by managing the guest residue composition, molecular weight, and ELP concentration [13,14]. This stimuli-responsive coacervation property is the driving force behind the use of ELPs in the field of protein purification [15] and drug delivery technology [6,16]. Even though the preparation of thermoresponsive hydrogels of ELPs is of considerable interest, there has been limited success in the practical use of this application, because ELPs tend to macroscopically phase-separate from a solution above *T*_t_ rather than form a gel as a continuous phase. Olsen et al. reported the formation of thermoresponsive hydrogels from ELPs (20 wt% in water), in which the third Gly in the pentapeptide repeating unit was substituted with Ala [17]. This substitution slowed the ELP’s dynamics above *T*_t_, thus enabling the formation of kinetically arrested and phase-separated nanostructures.

Modular protein polymers containing ELPs as building units have attracted increasing attention as a means of fine-tuning the self-assembly behavior of these polypeptides [5]. Conticello et al. biosynthesized amphiphilic ELP block copolymers with different temperature responses between the two blocks [18]; these copolymers were capable of forming nanoparticles with a narrow size distribution in water. Chilkoti, Rubinstein, and coworkers developed a theoretical model for the micellization of amphiphilic ELP block copolymers, where the relationships between the sequence and micellar structure were shown [19]. Chaikof et al. reported on reversibly changing the size of the micelles above and below *T*_t_; these micelles had been formed from ABA-type triblock ELPs, in which hydrophobic blocks (A) flanked the central hydrophilic block (B) [20]. Rodríguez-Cabello et al. reported on vesicle formation from a BAB-type ELP block copolymer [21]. The formation of thermoresponsive hydrogels was also reported from diblock [22] and ABA triblock [23] copolymers above 5 wt%. The modular polypeptide, EP20-24-24, which consisted of three hydrophobic domains of a monomer elastin interspersed by two crosslinking domains, formed a gel at relatively low concentrations (0.34 wt%) [24]. The fusion proteins of ELPs with silk-like polypeptides have been vigorously investigated for biomedical applications [25,26,27]. Dual self-assembling mechanisms that were derived from the hydrophobic associations of ELP and hydrogen bonding formation from silk-like sequences made the resultant gel stiffer than the corresponding ELP hydrogel [27].

**Figure 1:**
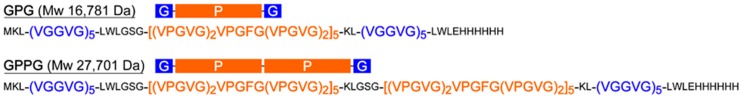
Amino acid sequences of **GPG** and **GPPG.**

Our group has developed a novel class of “double-hydrophobic” ELP block copolymers, named **GPG** [28,29,30,31,32], which was inspired by the non-homogeneous distribution of glycine-rich and proline-rich hydrophobic domains in monomer elastin [33]. **GPG** contains the basic sequence (VGGVG)_5_-(VPGXG)_25_-(VGGVG)_5_, where X is V (80%) or F (20%) (F: Phe) to tune the *T*_t_ at around room temperature (Figure 1). The glycine-rich (VGGVG)_5_ and the proline-rich (VPGXG)_25_ both dehydrate at higher temperatures; however, they form distinct secondary structures, namely, β-sheet and β-turn structures, respectively. In water above 37 °C, **GPG** initially assembles to form nanoparticles that are rich in β-turn structures that connect to generate beaded nanofibers with the formation of β-sheet structures between the nanoparticles [28,29,32]. The distinguishing feature of **GPG** over conventional ELPs is that the nanofibers are obtained as a stable colloidal dispersion without forming precipitates; therefore, they can be easily coated on substrates through the drying process [29]. Furthermore, various functional amino acid motifs, including crosslinkable [30], cell binding [29], and silver binding [31] sequences, can be fused to the C-terminus of **GPG** without hampering its fiber-forming abilities, thus demonstrating the robust self-assembling characteristics of this block sequence. However, it has never been clarified whether the block length exerted influence on the self-assembly of this double-hydrophobic polypeptide, despite the fact that the block length is an important parameter in the self-assembly processes of block copolymers [19,25,34]. This study focuses on the newly constructed **GPPG**, in which the proline-rich (VPGVG)_25_ is repeated twice via a short linker sequence (Figure 1). Differences in the self-assembled nanostructures of **GPG** and **GPPG** were examined through the analysis of the nanofiber formation while using circular dichroism (CD) spectroscopy, atomic force microscopy (AFM), and transmission electron microscopy (TEM). The rheological characteristics of the fiber dispersions of these double-hydrophobic polypeptides were investigated for the first time, which revealed their solid-like behavior, even at extremely low concentrations (0.034 wt%).

## 2. Results

### 2.1. Synthesis of GPPG

**Figure 2:**
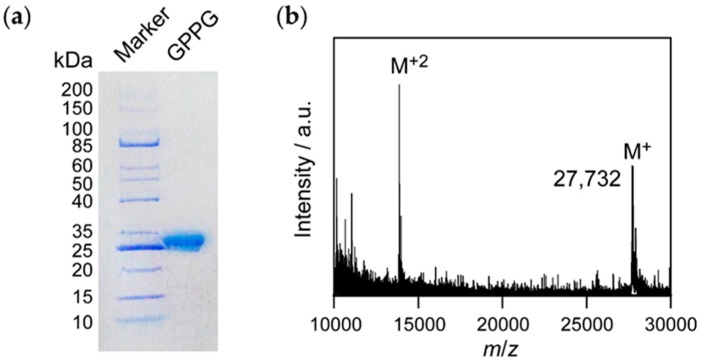
(**a**) Sodium dodecylsulfate polyacrylamide gel electrophoresis (SDS-PAGE) (12.5% tris-tricine gel) and (**b**) matrix-assisted laser desorption/ionization time of flight mass spectrometry (MALDI-TOF-MS) spectrum of the purified protein.

The theoretical molecular weight of **GPPG** is 27,701 Da. A clear band that corresponded to **GPPG** was observed between 25 and 35 kDa during sodium dodecylsulfate polyacrylamide gel electrophoresis (SDS-PAGE, Figure 2a). There was another band at 25 kDa that was just below the band corresponding to **GPPG**. This was presumably attributed to SlyD (molecular weight: 20.8 kDa), a histidine-rich protein derived from *E.coli* [35]. Matrix-assisted laser desorption/ionization time of flight mass spectrometry (MALDI-TOF-MS) showed the presence of a peak at 27,732 Da (Figure 2b). However, the peak originating from SlyD was not detected in the MS spectrum. These results indicated that **GPPG** was successfully expressed and purified as the main product. The successful synthesis of **GPG** was reported in our previous study [28].

### 2.2. Self-Assembly of GPG and GPPG

**Figure 3:**
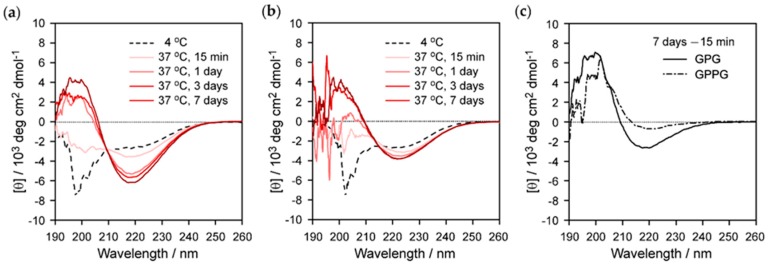
Circular dichroism (CD) spectra of (**a**) **GPG** and (**b**) **GPPG** at 20 μM in water. (**c**) CD difference spectra of **GPG** and **GPPG** obtained by subtracting the spectra acquired at 37 °C, 15 min. from those acquired at 37 °C, 7 days in (**a**) and (**b**). The data for **GPG** spectra at 37 °C in (**a**) and for the difference spectrum of **GPG** in (**c**) are adopted from our previous report with permission from ref. [29]. Copyright 2017, Wiley.

Figure 3a,b show the CD spectra of **GPG** and **GPPG** (20 μM), respectively. It should be noted that the spectra of **GPPG** between 190‒197 nm are inaccurate, because of the low signal-to-noise ratio due to relatively high weight fraction of **GPPG** (0.055 wt%). At 4 °C, an intense negative band at around 200 nm, with a negative shoulder at around 220 nm, was observed for both **GPG** and **GPPG**, which indicated that they adopted predominantly disordered structures that contained some β-turn structures. Soon after increasing the temperature to 37 °C, the intensity of a negative band at 200 nm decreased and the one at 220 nm increased, which showed the formation of more β-turn structures. The spectrum of **GPG** noticeably changed with time at 37 °C; the intensity of the negative band seen at around 220 nm continuously increased over the course of seven days with slight blue shifts [28,29]. In addition, the band observed at around 200 nm became positive after one day, and its intensity increased with time. A similar change in spectra was observed for **GPPG**, whereas the intensities of the negative bands seen at around 220 nm between one and seven days were smaller than those of **GPG**. The CD difference spectra (that compared samples at 15 min. and seven days at 37 °C) highlighted the nature of this transition; positive peaks observed at around 200 nm and negative peaks at 218 nm signaled the development of β-sheet structures with time [29]. The intensity of the molar residue ellipticity of **GPG** was higher than that of **GPPG**, which indicated that the β-sheet content per residue was higher for **GPG**. This result is compatible to the fact that the content of the β-sheet-forming VGGVG repeating units per molecule was higher in **GPG** than in **GPPG**.

**Figure 4:**
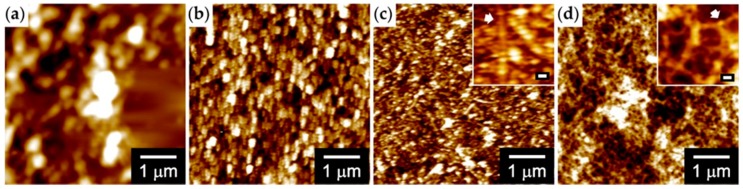
Atomic force microscopy (AFM) images of the assembled structure of **GPPG** at (**a**–**c**) 20 μM (0.055 wt%), and (**d**) 12 μM (0.034 wt%). The images were obtained after (**a**) 15 min., (**b**) 3 days, and (**c** and **d**) 7 days at 37 °C. The scale bars in insets in (**c**) and (**d**) are 50 nm. The arrows show the beaded morphology of the nanofibers.

AFM was used to monitor the time-dependent morphological change in **GPPG** (20 μM, 0.055 wt%) (Figure 4a–c). **GPPG** followed a similar self-assembly pathway to that observed for **GPG** in our previous study [28]. Spherical particles of various sizes were observed 15 min. after the temperature was increased to 37 °C (Figure 4a). The size of particles became more homogeneous after three days and then smaller after seven days, and they were organized as beaded nanofibers (Figure 4c) with an average fiber diameter of 56 ± 16 nm, which was larger than that of **GPG** (42 ± 8 nm at 20 μM, 0.034 wt%) [29]. The nanofibers formed from 12 μM (0.034 wt%) **GPPG** were also observed (Figure 4d) for comparison of the fiber morphology of **GPG** and **GPPG** at the same mass fraction. Beaded nanofibers with a diameter of 55 ± 11 nm were observed, which indicated that the concentration of **GPPG** (0.034 or 0.055 wt%) did not affect the morphology of the nanofibers. It should be noted that the diameter of nanofibers might be overestimated; the heights of nanofibers were measured to be around 10 nm in the AFM images. This is because of the relatively low lateral resolution due to the tip–sample convolution effect and/or deformation of nanofibers on the substrate.

**Figure 5:**
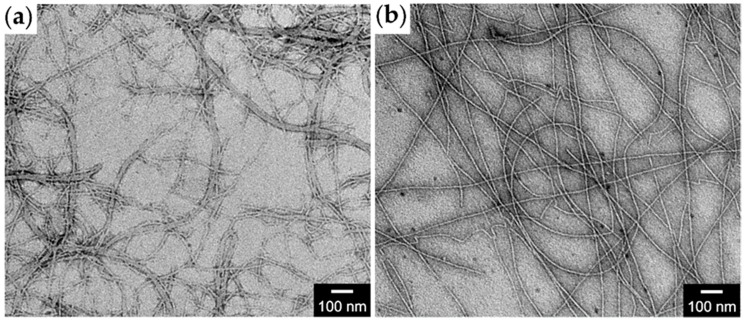
Transmission electron microscopy (TEM) images of nanofibers of (**a**) **GPG** and (**b**) **GPPG** at 0.034 wt%.

The nanofibers of **GPG** and **GPPG** (0.034 wt%) were observed with TEM to obtain higher-resolution images (Figure 5). **GPG** formed branched nanofibers ca. 5–30 nm in diameter (Figure 5a). Some nanofibers laterally assembled to form bundles. The beaded nanostructure of the nanofibers was not observed probably because of progressive dehydration under vacuum conditions, in contrast to the results from the AFM observation in our previous study [29]. On the other hand, more homogeneous, less branched nanofibers ca. < 10 nm in diameter were observed from **GPPG** (Figure 5b). It was difficult to estimate the length of both nanofibers, because they were long enough to protrude from the field of view in the TEM images. A lower magnification image of **GPPG** showed that the fiber length was at least 5 μm (Figure S1).

### 2.3. Rheology of Nanofiber Dispersions

**Figure 6:**
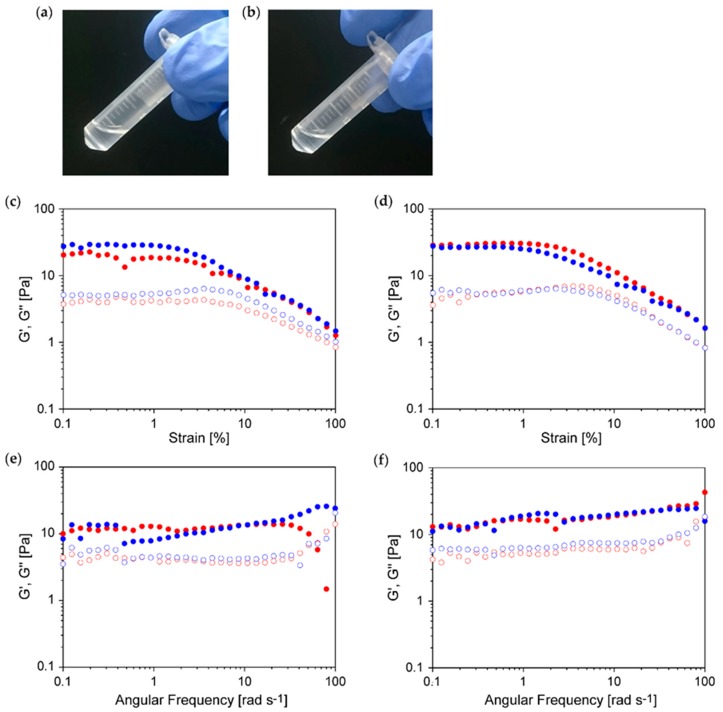
(**a**,**b**) Photographs of the nanofiber dispersions and (**c–f**) the rheological properties of (**a**,**c**,**e**) **GPG** and (**b**,**d**,**f**) **GPPG** at 0.034 wt%. Graphs c and d show the strain sweep (1 Hz), whereas graphs e and f show the frequency sweep (1% strain). The sample was collected twice from a nanofiber dispersion, and both readings were conducted independently. The red and blue circles correspond to the first and second fractions, whereas the solid and open circles represent G′ and G”, respectively.

The nanofibers of **GPG** and **GPPG** were obtained as optically clear dispersions with no precipitates (Figure 6a,b), which prompted us to evaluate the rheology of these fiber dispersions. Figure 6c–f show the dynamic rheological characteristics of the nanofiber dispersions at 0.034 wt%. The sample was collected twice from a nanofiber dispersion and, in both cases, readings were conducted independently to confirm reproducibility. The rheological behavior observed for **GPG** and **GPPG** fiber dispersions was similar. Both moduli were shown to decrease at above 2% strain, even though strain sweep measurements revealed that the storage modulus (G′) and the loss modulus (G”) were relatively constant (between 0.1% and 2% strains) (Figure 6c,d). This was indicative of a transition from a linear to non-linear viscoelastic regime. Frequency sweep measurements showed that G′ was greater than G” over a wide range of frequencies, highlighting the solid-like behavior of these dispersions (Figure 6e,f). Fluctuations between the samples were observed for **GPG**, and there was an anomalous drop for G′ above 30 rad s^−1^ only for the first fraction.

## 3. Discussion

In this study, a new double-hydrophobic ELP derivative, **GPPG**, was successfully constructed. **GPPG** had a proline-rich sequence (VPGXG)_25_, which was double the length of **GPG** and with a short linker sequence as the middle block. **GPPG** dissolved in 4 °C water to form the disordered structures that were seen in the CD spectrum (Figure 3b). In general, the increase in molecular weight of (VPGXG)_n_ resulted in a decrease in *T*_t_ [13,14]. Thus, the *T*_t_ of **GPPG** (20 μM) was estimated to be between 4 and 16 °C, which was the reported *T*_t_ of 20 μM **GPG**.

**Figure 7:**
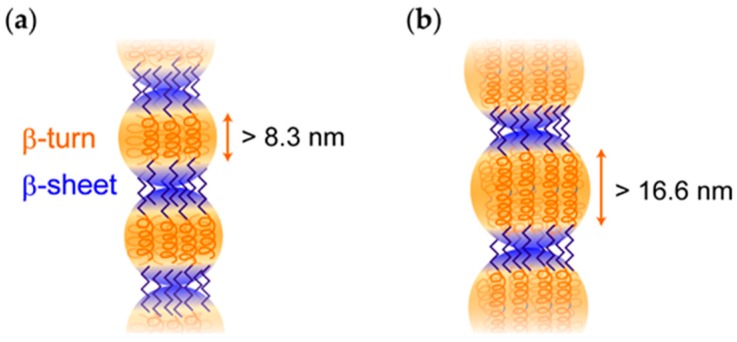
Plausible structural model for beaded nanofibers of (**a**) **GPG** and (**b**) **GPPG**.

**GPPG** underwent a self-assembling pathway that was similar to that of **GPG** via the formation of the nanoparticles above the *T*_t_ followed by maturation into nanofibers through the subsequent formation of β-sheet structures. The structural differences that were found in the resultant **GPG** and **GPPG** nanofibers were related to the content of the β-sheet structures and the diameter of the nanofibers. The β-sheet content per residue was **GPG** > **GPPG** (Figure 3c), which was expected from the sequences in which the content of β-sheet forming VGGVG repeating units per molecule was higher for **GPG**. The average diameters of the nanofibers, as determined via AFM, were 55 ± 11 nm (**GPPG**) > 42 ± 8 nm (**GPG**) at 0.034 wt%. A structural model for the nanofibers of **GPG** was proposed in our previous work (Figure 7a), in which the **GPG** molecules were shown to align with each other to form nanoparticles that were connected by interdigitated β-sheet structures [28]. We have proposed this model based on (1) the beaded morphology of nanofibers, (2) no spectrum change of Congo red and thioflavin T (dyes sensitive to cross-β structures) in the presence of the nanofibers [32], and (3) the presence of oligohistidine tag (His tag); the hydrophilic His tags should induce the adjacent (VGGVG)_5_ segments to locate near the surface of the particle [28]. Given that the proline-rich (VPGXG)_25_ segment formed ideal β-spiral structures, as previously proposed by Urry et al. [13], the length of the segment was estimated to be ca. 8.3 nm due to the β-spiral structure, which possessed a spiral pitch of 1 nm, contained three VPGXG pentapeptide units per turn. However, recent molecular dynamics simulation showed that the β-spiral model was too ideal and that an ELP, in this case (VPGXG)_n_, contained predominantly random coil structures in which each repeat was independently capable of transiently sampling β-turn and polyproline type II (PPII) structures [36,37]. Taking these into account, it is understandable that the length of (VPGXG)_25_ segment, in fact, should be longer than the 8.3 nm expected from the β-spiral model. Figure 7b shows a plausible structure for **GPPG** where the same model as **GPG** was applied. The particle size increased because of the doubling in size of the central segments when compared to that of **GPG**. This model could be used to account for the larger fiber diameter (13 nm larger than that of **GPG**) of **GPPG** while assuming that the fiber diameter was roughly same as the particle diameter. Another difference that was found in the TEM observations was the branching and lateral associations between the **GPG** nanofibers, whereas no branching or association was observed for the **GPPG** nanofibers (Figure 5). The association between **GPG** nanofibers could be a result of smaller fiber diameter. The intrinsically hydrophobic nature of these nanofibers meant that they showed a predilection for assembly in order to reduce the interfacial area between the nanofiber and the water molecules. The formation of branchless nanofibers from **GPPG** was unexpected, since there is greater freedom in the molecular configuration of **GPPG** due to the presence of a flexible linker sequence KLGSG between the VPGXG repeating units, which could rather cause branching of nanofibers. Nevertheless, branchless nanofibers were formed from **GPPG**, because there might be the π-π stacking interactions between the aromatic rings from F guest residues in VPGXG repeating unit, which facilitate the alignment of (VPGXG)_25_ segments. In addition, molecular exchange kinetics between nanofibers should be much smaller for **GPPG** due to its longer chain length [38], which reduces a frequency of dissociation of **GPPG** molecules from the nanofibers that triggers fiber branching.

Despite the aforementioned differences in nanofiber structures, **GPG** and **GPPG** behaved similarly in rheological experiments except for the poor reproducibility in frequency sweep measurements for **GPG** (Figure 6). The steep drop in G’ that was seen at higher frequencies, which was most probably due to a change in the nanostructure of the dispersion, was detected in other nanofiber dispersion systems [39]. **GPG** nanofibers may be locally aggregated in a dispersion, as suggested by the TEM observation (Figure 5a), which causes a slight sample-to-sample variation in the rheological readings.

The self-assembly system of the current double-hydrophobic polypeptides has some similarities with that of EP20-24-24 [24] and the ELP with silk-like sequences [26,27], in terms of the initial hydrophobic association followed by maturation into the nanofibers through β-sheet formation. Nevertheless, the solid-like behavior of the dispersion at this extremely low concentration (0.034 wt%) has never been reported for any ELP-based polymer. The viscoelastic properties that were presented in this paper were also uncommon among other fiber dispersion systems to the best of our knowledge. For example, Hsiao, Söderberg, and coworkers reported on the mechanistic behavior of charged cellulose nanofibers (CNF) in aqueous systems [39]. The dispersion behaved solid-like (G’ > G”) at 0.1 wt% nanofiber concentration with G’ of ca. 1 Pa, while it behaved liquid-like (G’ < G”) at 0.03 wt% with G’ of ca. 0.02 Pa. On the other hand, our data show that dispersions of double-hydrophobic ELPs were solid-like at 0.034 wt% with G’ of ca. 10 Pa.

The rheological properties of fiber dispersions have been described based on the concept of fiber flocculation [40]. Flocculation takes place above a certain concentration threshold, where the fibers initiate continuous contact in a network structure. Whereas, the number of contact points, the stiffness of the fibers, and the friction between fibers controls the mechanistic character of the dispersion dictated by fiber flocculation, the primary controlling mechanism is the number of contact points [39,41]. To characterize this, Kerekes and Schell defined a crowding factor, *N*, which represented the number of fibers within the rotational sphere of influence of a single fiber [41]. The values for *N* were calculated from the volume fraction of fibers, *ϕ*; fiber length, *L*; and, fiber diameter, *D*, while using the following equation:

(1) $$N=\frac{2}{3}\phi {\left(\frac{L}{D}\right)}^{2}$$

Continuous interfiber contact is expected when *N* > 60. Hsiao et al. found that the *N* value was closely correlated with the dynamic moduli of the dispersions of charged CNF [39,42]. The dispersion exhibited a fluid behavior (G” > G’) when *N* < 16, a soft gel behavior (G” ≈ G’) when 16 < *N* < 60, and a hard gel behavior (G’ > G”) when *N* > 60. For example, *N* was calculated to be < 16 for the liquid-like dispersion of charged CNF (0.03 wt%), the aspect ratio (L/D) of which was ca. 100 [39]. The lengths of nanofibers (*L*) of **GPG** and **GPPG** were calculated at *N* = 16 and 60, respectively, by using the relationships between *N* and the nanofiber aspect ratio (Table 1). The *L* can be estimated to be > 22 μm and > 28 μm for **GPG** and **GPPG**, respectively, because *N* values are expected to be > 60 because G’ > G” for **GPG** and **GPPG** dispersions at 0.034 wt%. In the present findings, the rheological characteristics are similar between **GPG** and **GPPG**, regardless of their structural differences in terms of β-sheet content and the degree of fiber branching, which should affect the stiffness of the fibers and the friction between fibers. Nevertheless, these structural differences between **GPG** and **GPPG** were not reflected in the rheological characteristics. This fact also supports the formation of sufficiently long nanofibers with high aspect ratio as the primary mechanism that governs the rheological behavior.

In conclusion, this study showed the sequence–structure–property relationships of elastin-like, double-hydrophobic polypeptides, **GPG** and **GPPG**. **GPG** has a block sequence of (VGGVG)_5_-(VPGXG)_25_-(VGGVG)_5_, whereas the central sequence (VPGVG)_25_ is repeated twice in **GPPG**. Polypeptides both self-assembled to form nanofibers in water at 37 °C. **GPPG** gave thicker nanofibers with smaller β-sheet content compared to **GPG**. The dispersions of both polypeptides showed similar dynamic viscoelastic properties and exhibited solid-like behavior at extremely low polypeptide concentration (0.034 wt%) with storage modulus G’ > loss modulus G” over a wide range of angular frequencies. This is most probably due to the formation of sufficiently high-aspect-ratio nanofibers from these polypeptides that leads to the flocculation of nanofibers in the dispersion. The formation of self-supportive hydrogels are expected at higher polypeptide concentrations, although the present polypeptide dispersions could not support their macroscopic structures in tilted vials due to a G’ value of ca. 10 Pa [43]. The findings revealed in this study will provide useful insights in the design of polypeptide-based scaffold and artificial ECMs in the future.

## 4. Materials and Methods

### 4.1. Plasmid Construction

The plasmid pET22b(+)-GPG encoding GPG polypeptide was previously constructed [28]. A gene fragment encoding for the proline-rich sequence [(VPGVG)_2_VPGFG(VPGVG)_2_]_5_ (P) was amplified via polymerase chain reaction (PCR) while using pET22b(+)-GPG as the template. PCR amplification modified the 3’ end of the P gene by introducing a *Bam*HI restriction site. The PCR product was isolated via *Bam*HI digestion and inserted into pET22b(+)-GPG at the *Bam*HI site to give pET22b(+)-GPPG. Restriction mapping and DNA sequencing analysis verified the gene sequence.

### 4.2. Polypeptide Expression and Purification

Plasmids pET22b(+)-GPG and pET22b(+)-GPPG were each transformed into the *E.coli* BLR (DE3) strain. The polypeptides were expressed, as previously reported [29], and they were purified using Ni-NTA affinity chromatography since both contained His tags at their C-termini. The purity of the polypeptides was confirmed via SDS-PAGE and MALDI-TOF-MS while using an AXIMA-CFR Plus spectrometer (Shimadzu, Kyoto, Japan). The polypeptides were stored as lyophilized powder at −20 °C prior to use.

### 4.3. Sample Preparation

The polypeptide powders were dissolved in cool water and then agitated at 4 °C overnight. Concentrations of the polypeptides were determined by measuring the absorbance at 280 nm while using a Nanodrop 2000 UV-visible spectrometer (Thermo Fisher Scientific, Wilmington, DE, USA). Adequate amounts of milliQ water were added to adjust their concentrations to 20 μM (0.034 wt% for GPG and 0.055 wt% for GPPG) or 12 μM (0.034 wt% for GPPG). The sample solutions were kept on ice to prevent any undesired aggregation prior to performing the experiments.

### 4.4. Characterization of Nanofiber Structures

The CD spectra were obtained with a J-820 spectrometer (Jasco, Tokyo, Japan). The readings were collected from 190 to 260 nm. The final spectra were acquired by subtracting the spectrum of the blank solvent. The data are expressed, as follows: ellipticity/[path length (cm) × concentration (mol/L) × number of residues × 10].

AFM was carried out while using an MFP-3D Origin^TM^ AFM, Oxford Instruments plc, U.K., via the tapping mode in air at room temperature. Scans were performed with a Si cantilever (OMCL-AC240TS, Olympus, Japan) at a rate of 1.0 Hz. The samples were each cast on a freshly cleaved mica substrate and allowed to dry at 37 °C. Fiber diameters were quantified by measuring widths of nanofibers by randomly picking 100 fibers in the AFM images using ImageJ.

TEM was carried out using JEOL JEM-2100plus at an accelerating voltage of 200 kV. The samples were applied to a carbon-coated grid (Cu 100 mesh), stained with phosphotungstic acid, and the excess solution was wiped away using blotting paper.

### 4.5. Rheological Measurements

The rheological measurements were carried out using an Anton Paar MCR302 rheometer with a 1° cone-and-plate configuration (25 mm diameter). The experiments were performed at a constant temperature of 37 °C, which was controlled using an integrated Peltier system. A solvent trap cover was used to keep the sample hydrated. The sample was transferred to the stage while using a micropipette.

## Figures and Tables

**Table 1:** Estimation of nanofiber lengths of **GPG** and **GPPG**.

	*N*	*Φ^a^*	*D^b^* [nm]	*L* [nm]
**GPG**	16	3.4 × 10^‒4^	42	11 × 10^3^
	60	3.4 × 10^‒4^	42	22 × 10^3^
**GPPG**	16	3.4 × 10^‒4^	55	15 × 10^3^
	60	3.4 × 10^‒4^	55	28 × 10^3^

*^a^* 1.0 g cm^−3^ was assumed as the density of the polypeptide nanofibers. ^*b*^ Determined by AFM.

## Supplementary Materials

Supplementary materials can be found at https://www.mdpi.com/1422-0067/20/24/6262/s1.

## Abbreviations

ELP	Elastin-Like Polypeptide
ECM	Extracellular Matrix
CD	Circular Dichroism
AFM	Atomic Force Microscopy
TEM	Transmission Electron Microscopy
SDS-PAGE	Sodium dodecylsulfate polyacrylamide gel electrophoresis
MALDI-TOF-MS	Matrix-assisted laser desorption/ionization time of flight mass spectrometry
CNF	Cellulose Nanofibers
