# Supplementary material for: Rheology of Dispersions of High-Aspect-Ratio Nanofibers Assembled from Elastin-Like Double-Hydrophobic Polypeptides
Source: Int J Mol Sci. 2019 Dec 12;20(24):6262. doi: 10.3390/ijms20246262 (PMC6940774; doi:10.3390/ijms20246262)
Supplement: Supplementary file 1 [file ijms-20-06262-s001.pdf]

Supplementary Material

Rheology of dispersions of high-aspect-ratio nanofibers assembled from elastin-like double-hydrophobic polypeptides

Ayae Sugawara-Narutaki [1,*], Sawako Yasunaga [2], Yusuke Sugioka [1], Duc H. T. Le [1,†], Issei Kitamura [3], Jin Nakamura [1], and Chikara Ohtsuki [1]

[1] Department of Materials Chemistry, Graduate School of Engineering, Nagoya University, Furo-cho, Chikusa-ku, Nagoya 464-8603, Japan
[2] Department of Crystalline Materials Science, Graduate School of Engineering, Nagoya University, Furo-cho, Chikusa-ku, Nagoya 464-8603, Japan
[3] Department of Molecular and Macromolecular Chemistry, Graduate School of Engineering, Nagoya University, Furo-cho, Chikusa-ku, Nagoya 464-8603, Japan

* Correspondence: ayae@chembio.nagoya-u.ac.jp
† Present Addresses: Department of Biomedical Engineering, Eindhoven University of Technology, P.O. Box 513 (STO 3.25), 5600MB Eindhoven and Department of Biochemistry, Radboud Institute for Molecular Life Sciences (RIMLS), Radboud University Medical Center, Geert Grooteplein 28, 6525 GA Nijmegen, The Netherlands

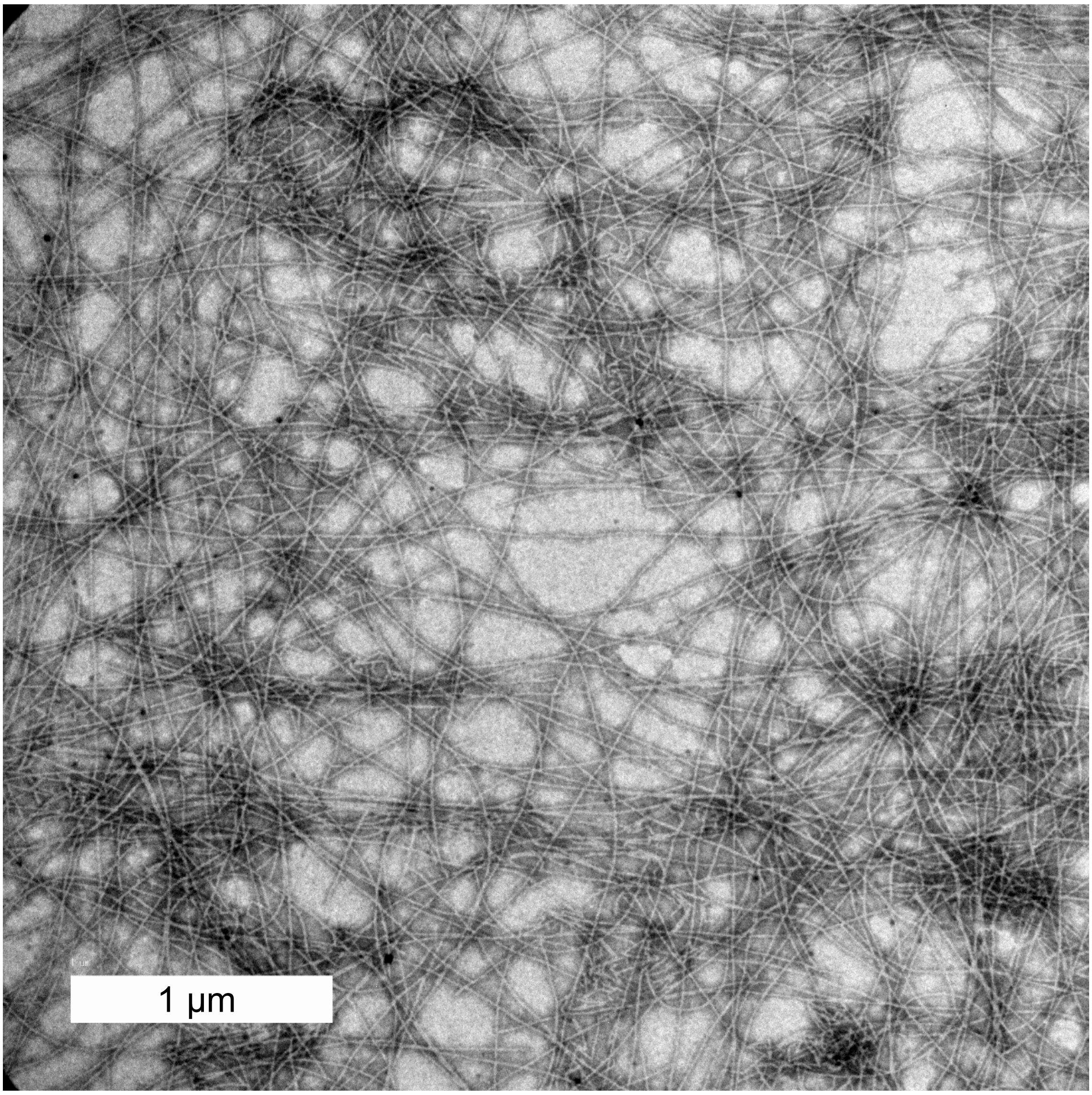

Figure S1. Transmission electron microscopy image of nanofibers of **GPPG** at 0.034 wt%. The sample was negatively stained with phosphotungstic acid.
